# Psychosocial Perspective on Problem Gambling: The role of Social Relationships, Resilience, and COVID-19 Worry

**DOI:** 10.1007/s10899-022-10185-9

**Published:** 2023-01-09

**Authors:** Anu Sirola, Jussi Nyrhinen, Terhi-Anna Wilska

**Affiliations:** Department of Social Sciences and Philosophy, University of Jyväskylä, Jyväskylä, Finland

**Keywords:** COVID-19, Gambling, Loneliness, Problem gambling, Resilience, Social support

## Abstract

The COVID-19 pandemic has amplified several psychosocial risks and problem behaviors among vulnerable individuals. Given that gambling has high addictive potential, it is important to consider the underlying mechanisms of problem gambling. This study examined psychosocial factors associated with pandemic-time problem gambling.

Cross-sectional data were gathered via an online survey of 18–75-year-old Finnish, Swedish, and British respondents (n = 2,022) who reported having gambled at least occasionally during the pandemic. Measures included problem gambling, loneliness, COVID-19 worry, social support, and psychological resilience. Control variables included gender, age, and education. Structural equation modeling was used as an analytical technique.

Loneliness was found to be associated with problem gambling. While COVID-19 worry was not directly associated with problem gambling, it predicted higher loneliness, which in turn was associated with problem gambling. Evidence was not found regarding the protective role of resilience or social support in problem gambling. However, social support was found to be associated with higher problem gambling severity. Male gender and younger age were associated with problem gambling.

The results bring insight into underlying vulnerabilities regarding problem gambling during the pandemic. More focus should be placed on the quality and sources of social support, as well as on how psychosocial risk and protective factors might work differently among different populations of gamblers.

## Introduction

Gambling is an activity characterized by wagering or betting mechanisms in which money or something of monetary value is at stake. Typical examples of gambling include lotteries, scratch cards, sports betting, and casino games. For most individuals, gambling is a harmless activity, but for others, gambling takes excessive forms. When gambling develops into excess, it brings various forms of emotional, social, and financial harm to individuals and their loved ones (Langham et al., 2015). In the 5th edition of *The Diagnostic and Statistical Manual of Mental Disorders* (DSM-5), excessive and pathological forms of gambling are recognized as “gambling disorder,” with classifications of mild, moderate, and severe (Sleczka et al., 2015). Gambling disorder is an example of behavioral addiction (i.e., addiction to a certain behavior, such as gambling) as opposed to substance-related addictions (Mann et al., 2016). However, both types of addiction share many central commonalities, such as salience, mood modification, and tolerance (Griffiths, 2005; Mann et al., 2016). Notably, problem gambling often co-occurs with substance-related addictions and mental disorders (Lorains et al., 2011).

Gambling is increasingly taking place online. During the first year of the COVID-19 pandemic, online gambling was promoted due to several restrictions on land-based gambling opportunities and the cancelation of sports events (Emond et al., 2022; Håkansson, 2020a). There were concerns in the early onset of the pandemic that online gambling and its excessive forms would increase in response to restrictions on other gambling opportunities (Brodeur et al., 2021). Contrary to expectations, problem gambling rates have not increased at the population level (Koós et al., 2022), and some studies have shown a decreasing trend in gambling over the course of the pandemic (Auer & Griffiths, 2022). However, there is growing evidence that gambling problems have been amplified among those who were actively involved in gambling prior to the pandemic (Brodeur et al., 2021; Emond et al., 2022; Håkansson, 2020b; Hodgins & Stevens, 2021).

The pandemic’s impact on gambling has been recognized as an important health hazard, as gambling has high addictive potential, and a pandemic might amplify the problems and addictive behaviors among already vulnerable individuals (Håkansson et al., 2020). Addictive behaviors often have underlying escapist motives, where such behavior is a response to stressful, burdening, or otherwise unideal life situations (Jouhki & Oksanen, 2022). Given that the pandemic has caused significant stress, uncertainty, and anxiety, excessive behaviors, such as high technology use, gambling, or substance use, have acted—for some individuals—as maladaptive ways of managing pandemic-related psychological distress and the restrictions of everyday life (Avena et al., 2021; Király et al., 2020). Moreover, as the pandemic has caused or worsened financial distress for many individuals, gambling might also have been perceived as a way to earn money easily (Emond et al., 2022; Price, 2020). Problem behaviors, such as excessive gambling and high alcohol use, also co-occurred during the pandemic (Emond et al., 2022; Håkansson, 2020b; Price, 2020). However, some individuals might be more susceptible to developing harmful habits and addictive behaviors during crisis situations, while others have healthier resources for dealing with unexpected and stressful situations.

While previous research has recognized many potential risk factors for problem gambling, such as male gender, young age, impulsivity, parental gambling problems, and comorbid mental disorders (e.g., Dowling et al., 2017; Sharman et al., 2019), less focus has been placed on psychosocial factors and potential protective factors, particularly during the pandemic. This study examines psychosocial factors associated with problem gambling among adult gamblers aged 18–75, utilizing data from Finland, Sweden, and the United Kingdom collected after the first year of the COVID-19 pandemic. In these culturally relatively similar European countries, gambling is a popular activity, and gambling problems are relatively common (Calado & Griffiths, 2016). Additionally, problem gambling and related problems have been reported during the COVID-19 pandemic in these countries (Håkansson, 2020a, b; Savolainen et al., 2022a; Sharman et al., 2021). Regarding pandemic safety measures, these countries adopted different strategies over the course of the pandemic. The UK adopted stricter and legally enforced measures, such as lockdowns, whereas in Sweden, safety measures were more lenient and mostly recommendation based. Finland fell in between, utilizing both legally enforced and recommendation-based measures. However, despite the differences in pandemic strategies, our preliminary analyses did not reveal significant country differences; thus, our analytical focus was not on cross-country comparisons. Rather, our aim was to create a theoretical model of psychosocial factors associated with problem gambling by utilizing multinational data gathered during the COVID-19 pandemic.

Our study makes several contributions. First, we answer a call for research to better understand pandemic-time gambling behavior and the underlying factors related to problem gambling (Brodeur et al., 2021; Hodgins & Stevens, 2021). Second, this study contributes to the literature regarding psychosocial factors in problem gambling. Drawing from our theoretical perspective, we approach loneliness and COVID-19 worry as potential risk factors and resilience and social support as potential protective factors for pandemic-time problem gambling. In doing so, we also address the need to explore potential protective factors in problem gambling, which has received relatively less attention and mixed results in previous research, particularly among adult gamblers (Bush et al., 2021; Scholes-Balog et al., 2015). Third, the results help us understand why some gamblers are more susceptible to developing gambling problems during crisis situations and which factors might be beneficial in protecting gambling behavior from developing into excess. Given that gambling has high addictive potential and that the pandemic has amplified vulnerabilities, it is important to understand how unforeseen pandemic situations and future crises might manifest in problem behaviors.

## Theoretical Background and Hypotheses

### The role of Social Support and Loneliness in COVID-19 Worry

Meaningful social connections and a sense of belonging are important assets for general well-being, but the protective role of social support is often emphasized during stressful or adverse situations (Taylor, 2011). Social support refers to the perception of being cared for by meaningful others, and this support can take specific forms, such as emotional or informational support (Taylor, 2011). Sources of social support may vary from offline (e.g., family, relatives, peers, colleagues) to online peer support communities and other networks (e.g., Ali et al., 2015; Savolainen et al., 2019; Taylor, 2011).

Loneliness is an aversive subjective experience of social disconnection and perceived deficiency in one’s relationships (Heinrich & Gullone, 2006; Perlman & Peplau, 1981; Weiss, 1973), and it concerns individuals in all age groups (Qualter et al., 2015). During the COVID-19 pandemic, social distancing and stay-at-home orders were common safety measures taken to curb the spread of the virus. Thus, social isolation and loneliness were common experiences during the pandemic (Liu et al., 2020). Loneliness has been associated with poorer mental health, such as anxiety and depression (MacDonald et al., 2022), while perceived social support has seemed to protect against mental distress during the pandemic (Liu et al., 2020).

Even though loneliness and social support are closely related, they are distinct concepts. As Luchetti et al., (2020) noted, even when physically isolated, perceived social support and a sense of being together during a crisis might also buffer against loneliness and related negative consequences. For example, digital technologies have allowed people to keep in touch with loved ones and to maintain other social connections during pandemic-imposed isolation and quarantines, which might have buffered against excessive loneliness. Therefore, we tested the following hypothesis:

H1: Social support is negatively associated with loneliness.

The COVID-19 pandemic and related safety measures, such as social distancing and quarantines, have widely affected mental health and have caused negative emotions for many individuals (Serafini et al., 2020). Given that the coronavirus is highly contagious and has caused a high number of deaths around the world, the pandemic has also caused significant worry over one’s own health and the health of loved ones (Son et al., 2020). Indeed, excessive worry and fear of getting the infection or infecting loved ones have been among the most typical psychological reactions to the pandemic (Serafini et al., 2020).

Loneliness is often accompanied by poor quality of life and negative emotions (Heinrich & Gullone, 2006; MacDonald et al., 2022). Pandemic research also suggests that negative emotions concerning the pandemic, such as rumination, worry, or anxiety, are particularly prevalent among lonely individuals (Arslan et al., 2022; Hoffart et al., 2020; Okruszek et al., 2020). Thus, we tested the following hypothesis:

H2: COVID-19 worry is positively associated with loneliness.

### The role of Social Support and Loneliness in Resilience

Some individuals have more capacity and resources to deal with adverse situations in life. Psychological resilience refers to the ability to not only cope with stress and negative life events but also recover after crises (Bonanno, 2004; Connor & Davidson, 2003; Fletcher & Sarkar, 2013). Resilience has been recognized as an important asset for protection from the pandemic’s adverse effects on mental well-being (Zhang et al., 2022).

Humans are inherently social; therefore, meaningful social relationships and a sense of belonging are vital to one’s physical and mental well-being (Baumeister & Leary, 1995; Deci & Ryan, 2000; Heinrich & Gullone, 2006). Insufficient social relationships are a risk factor for many problems and even premature mortality (Holt-Lunstad et al., 2010). The literature on resilience has recognized that social support plays an important role in cultivating resilience and protection from the negative health effects of stressful events (Cohen & Wills, 1985; Mancini & Bonanno, 2009; Zhang et al., 2022). Therefore, we propose the following hypotheses:

H3: Social support is positively associated with resilience.

H4: Loneliness is negatively associated with resilience.

### The role of Social Support and Loneliness in Problem Gambling

Social support from meaningful others is generally recognized as an asset that protects us from many risks and harms (Cohen & Wills, 1985; Kaakinen et al., 2018; Minkkinen et al., 2016). However, studies have yielded mixed results regarding the role of social support in gambling and problem gambling (Nordmyr & Forsman, 2020). A study by Savolainen et al., (2019) emphasized the role of offline support, showing that while offline support seems to protect from problem gambling, online support is a risk for problem gambling. By contrast, some studies have yielded mixed results regarding the role of offline peer support (Hardoon et al., 2004; Räsänen et al., 2016). It is also possible that high levels of social support indicate that a person is in distress and thus seeking more support. 

During the COVID-19 pandemic, social support and a sense of belonging might have been particularly important assets for well-being due to stay-at-home mandates and quarantines, thus protecting people from harmful and excessive behaviors. Given that social support is often recognized as a buffer against problem behaviors, the following hypothesis is worth testing:

H5: Social support is negatively associated with problem gambling.

Loneliness is a major stressor for well-being; it strains mental and physical health and increases the risk of premature mortality (Holt-Lunstad et al., 2010). Gambling, particularly online, typically takes place in isolation, as the gambler is physically alone, and the sole purpose is to win as an individual, as opposed to many digital games that involve a social aspect (Sirola et al., 2021). Lonely individuals are also prone to excessive online behaviors as a coping strategy to escape everyday problems and loneliness (Kuss et al., 2014). According to several studies, loneliness is a common experience among excessive gamblers (Khazaal et al., 2017; Sirola et al., 2019; Vuorinen et al., 2021). Particularly during the pandemic, excessive Internet use and consequent addictive behaviors, such as problematic gambling, might have acted as maladaptive ways to cope with loneliness. Therefore, we tested the following hypothesis:

H6: Loneliness is positively associated with problem gambling.

### The role of Resilience and COVID-19 Worry in Problem Gambling

Individuals with psychological resilience are able to cope with and adapt to stressful situations using functional coping strategies (Bonanno, 2004; Connor & Davidson, 2003; Fletcher & Sarkar, 2013). Excessive and addictive behaviors are often used as maladaptive coping strategies to deal with negative emotions, such as anxiety or stress (Griffiths, 2005). A meta-analysis by Hu et al. (2015) found that higher resilience was consistently associated with positive mental health. A study by Brailovskaia & Margraf (2022) suggested that positive mental health can increase one’s sense of control, which can further protect one from developing addictive behaviors.

The role of psychological resilience in problem gambling has received relatively little attention in previous research, and the available studies have yielded mixed results. There is evidence that resilience could protect people from problem gambling, particularly among youth (Lussier et al., 2007), but studies on adult problem gambling have not found evidence regarding the protective role of resilience (Mishra et al., 2019; Oei & Goh, 2015; Scholes-Balog et al., 2015). During the pandemic, experiencing mental health problems, such as depression or anxiety, was associated with gambling problems (Savolainen et al., 2022a; Sharman et al., 2021), and it is plausible to expect that those with higher resilience would have been less prone to engage in addictive behaviors during the pandemic due to more positive mental health. Therefore, we tested the following hypothesis:

H7: Resilience is negatively associated with problem gambling.

Even though physical gambling venues were widely closed due to pandemic safety restrictions, online gambling opportunities remained constantly available and easily accessible. High technology use has been a way to alleviate the stress and boredom related to the pandemic and its safety restrictions, such as social isolation (Király et al., 2020). For some individuals, problem gambling and other addictive behaviors are a maladaptive way to escape stressful life situations (Jouhki & Oksanen, 2022) and to cope with worry, anxiety, or stress related to the pandemic (Avena et al., 2021). Problem gambling is often accompanied by negative emotions and psychological distress, such as anxiety or depression (Barrault & Varescon, 2013; Oksanen et al., 2018). Anxiety and worry related to the pandemic can also increase mental health problems, which can manifest in problematic gambling (Savolainen et al., 2022a). Thus, we hypothesized the following:

H8: COVID-19 worry is positively associated with problem gambling.

### Control Variables

Age, gender, and education were added as control variables to the model. As with other behavioral addictions, problem gambling typically starts to develop during young adulthood, making adolescents and young adults the most vulnerable age groups (Derevensky et al., 2019; Hing et al., 2016; Salonen et al., 2020). Even though gambling and problem gambling concern predominantly young males (Dowling et al., 2017; Hing et al., 2016; Merkouris et al., 2016), recent research has shown an increasing trend in women developing problem gambling habits (Håkansson, 2020a). In some studies, low education has been recognized as a risk factor for problem gambling (Hing et al., 2016; Wu et al., 2014). Even though individuals in all age groups experience loneliness, it is particularly prevalent among adolescents and young adults (Qualter et al., 2015). During the COVID-19 pandemic, young adults and women reported higher levels of loneliness (Lee et al., 2020; Luchetti et al., 2020). Studies have also shown that older individuals are, in general, more resilient than younger ones (Gooding et al., 2012).

**Fig. 1:**
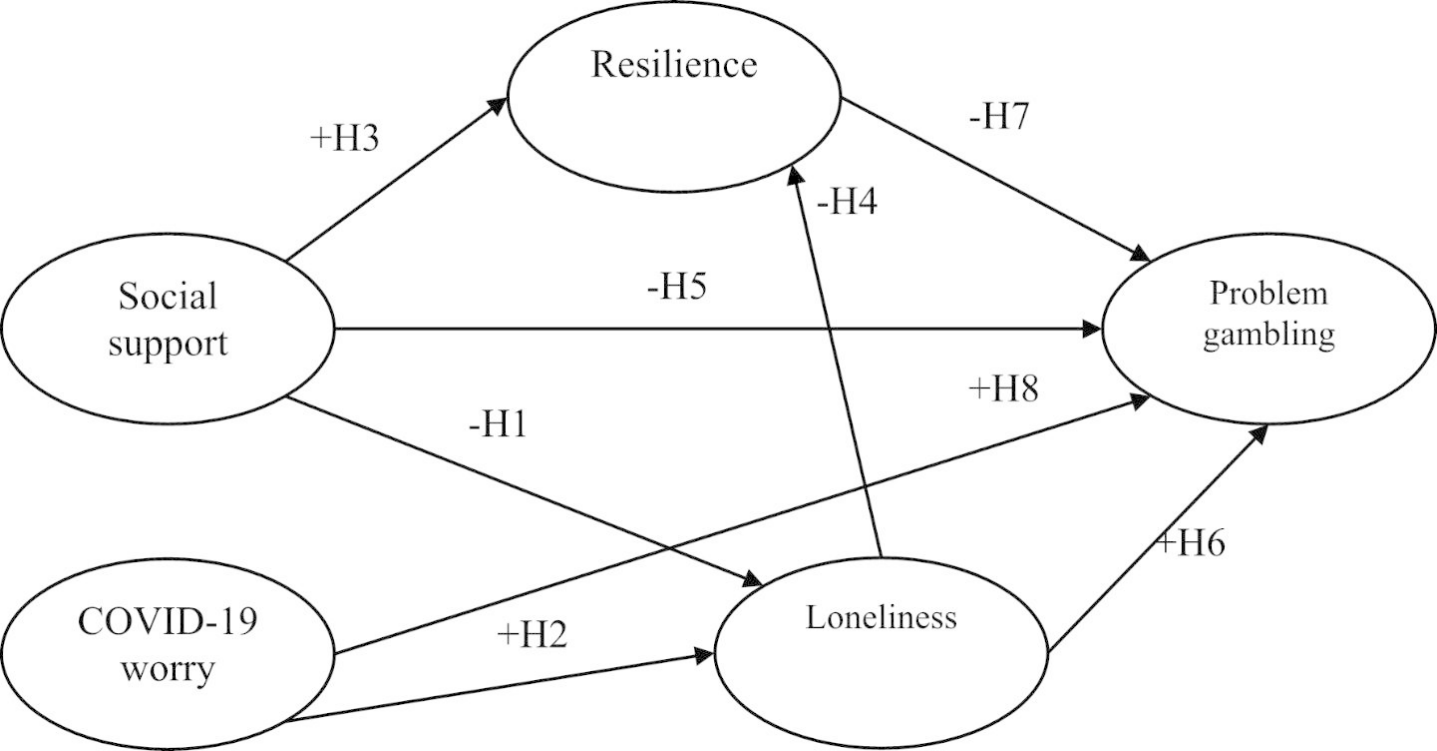
Theoretical Framework and Hypotheses

## Data and Methods

### Data Collection

A total of 2,022 18–75-year-old participants (female 45.1%; mean age = 43.00; SD = 15.24) were surveyed for this study, comprising participants from Finland (n = 709), Sweden (n = 714), and the UK (n = 599). The criterion for participating was that a respondent had gambled online or offline during the past 12 months of the pandemic, at least occasionally. Cross-national study data were gathered simultaneously from these countries in April 2021 during the COVID-19 pandemic using an anonymous online survey. The survey design and measures were similar in Finnish, Swedish, and English.

The web panel sample for the survey was gathered by a data provider company that recruited respondents from an online panel using a random sample from each country. Panel members were contacted in random order. The web panelists were volunteers who chose to respond to surveys according to their likes and interests. The panelists were also awarded prizes and compensation for their time and effort. Data collection was carried out according to the ethical standards stated by the Declaration of Helsinki. The respondents were informed about the aims of the survey, and their responses were fully voluntary. The respondents were also able to withdraw from the study at any point. The data did not include underaged participants, and the respondents’ identities were kept confidential.

### Measures

**Problem gambling** was measured with the Problem Gambling Severity Index (PGSI), which is a standardized measure developed for survey research to assess problematic gambling in non-clinical context (Ferris & Wynne, 2001; Holtgraves, 2009). The PGSI is one of the most frequently used instruments to assess problem gambling in population studies, and it has shown good psychometric properties across different countries (Abbott et al., 2018; Raisamo et al., 2015; Savolainen et al., 2022a). It has also been widely utilized in survey research during the pandemic to assess pandemic-time gambling problems (Sachdeva et al., 2022). The PGSI comprises items such as “Have you needed to gamble with larger amounts of money to get the same feeling of excitement?”. The answer options were provided on a scale from 0 to 3 (0 = never, 1 = sometimes, 2 = most of the time, or 3 = almost always). A 12-month timeframe was given to reflect pandemic-time problem gambling behaviors.

**Loneliness** during the pandemic was measured with a three-item loneliness scale, which is a short yet comparable version of the full UCLA loneliness scale, developed for survey research (Hughes et al., 2004). The scale has been widely used in studies assessing loneliness, and it has shown good psychometric properties across different countries (Johansson et al., 2021; Sirola et al., 2019; Vuorinen et al., 2021). It has also been widely utilized in survey research during the COVID-19 pandemic (Johansson et al., 2021; Liu et al., 2020; MacDonald et al., 2022). A three-part question was asked: “Thinking about the past year, how often have you felt 1) that you lack companionship, 2) left out, or 3) isolated from others?” The answer options were provided on a scale from 1 to 3 (1 = hardly ever, 2 = some of the time, or 3 = often). Higher scores indicate higher levels of loneliness. The original measure did not include a timeframe, but it was included here to measure pandemic-related loneliness (i.e., the past 12 months).

**Resilience** was measured using a shortened version of the Connor–Davidson resilience scale (CD-RISC) (Campbell-Sills & Stein, 2007; Connor & Davidson, 2003), which assesses psychological resources to cope with adverse situations, such as crises. The scale has shown good psychometric properties in studies assessing psychological resilience across different countries (Tourunen et al., 2021; Velickovic et al., 2020). The scale includes statements such as “I can deal with whatever comes my way,” with answer options provided on a scale from 0 to 4 (0 = not true at all; 4 = true nearly all of the time). Higher scores indicate higher levels of resilience.

**Social support** was measured using the Brief Form of the Perceived Social Support Questionnaire (F-SozU K-6). It is a valid and reliable measure for large-scale cross-cultural studies assessing perceived general social support, and it has shown good psychometric properties across diverse cultural contexts (Kliem et al., 2015; Lin et al., 2019). The scale includes items such as “If I am very depressed, I know who I can turn to,” with answer options provided on a scale from 1 to 5 (1 = not true at all; 5 = very true). Higher scores indicate higher levels of perceived social support.

**COVID-19 worry** was measured using the question “How worried are you about the impacts of coronavirus?” in terms of (1) the health of your loved ones, (2) your own mental well-being, and (3) the mental well-being of your loved ones. Similar questions have been used in previous studies to assess pandemic-related worry (e.g., Broos et al., 2022; Mónaco et al., 2022; Okruszek et al., 2020; Ranta et al., 2020) and other health-related worries in specific situations (Green et al., 2003). The answers were provided on a scale from 1 to 5 (1 = not worried at all; 5 = extremely worried). Higher scores indicate higher levels of worry.

**Sociodemographic controls** included respondents’ gender (male = 0, female = 1), age, and education.

### Common Method bias

Common method bias (CMB) was minimized through the following procedures (see Podsakoff et al., 2012). First, the order of the questionnaire items was mixed so that the items measuring the same dimension were not in a row. Second, we also switched response format between the scales. The purpose of these two procedures was to decrease the likelihood that respondents would base their subsequent answers to their earlier answers. Third, we minimized ambiguity of the statements that represented the items. The reason for this procedure was that respondents have difficulties to interpret the meaning of ambiguous statements and may therefore rely on systematic response patterns if items are not simple and specific. To disclose the possibility that the results could be interfered with by CMB, a common latent factor (CLF) test (Podsakoff et al., 2003) was conducted. In this test, CLF represents the common variation of the measurement model, and the purpose of the CLF test is to identify which item has potential to pervade more variance from CLF construct (Afthanorhan et al., 2021). The results of the CLF test show that CMB is unlikely to be an issue because the average method-based variance was 0.02. The average method-based variance is the average difference between the estimates without CLF is minus to estimates with CLF that should not be larger than 0.2, when CLF loadings are constraint to equal with all indicator variables (cf. Afthanorhan et al., 2021).

### Analysis Strategy

To test the conceptual model and proposed hypotheses, structural equation modeling with the maximum likelihood estimation method with bootstrapping was employed using AMOS 26 software.

The measurement scales comprised 18 items that involved 5 constructs (Table 1). The measurement model was designed to measure the following latent constructs: problem gambling, social support, loneliness, resilience, and COVID-19 worry.

The model fit was examined and found to show a good fit: (χ2(125) = 365.00, CMIN/DF = 2.92, IFI = 0.99, CFI = 0.99, TLI = 0.97, RMSEA = 0.03, 90% CI [0.03, 0.04], and SRMR = 0.03, RFI = 0.97). The validity of the measurement model and the unidimensionality of the constructed scales were tested with confirmatory factor analysis. The results of the reliability and validity of the measurement scales show that all component loadings were equal to or greater than 0.66.

**Table 1:** Item and Factor (Dimension) Statistics

**Indicator**	***M***	***SD***	**FL**
*Problem Gambling* (“Thinking about your gambling during the past year…”)			
Have you needed to gamble with larger amounts of money to get the same feeling of excitement?	0.66	0.90	0.82
Have you borrowed money or sold anything to get money to gamble?	0.58	0.92	0.86
Have you felt that you might have a problem with gambling?	0.66	0.94	0.85
Have people criticized your betting or told you that you had a gambling problem, regardless of whether or not you thought it was true?	0.56	0.87	0.85
Has your gambling caused any financial problems for you or your household?	0.59	0.92	0.86
*Social Support* (“Evaluate how well the following statements hold true in your case.”)			
If I need to, I can borrow something from friends or neighbors without any problems.	3.43	1.15	0.67
When I am sick, I can ask friends/relatives to handle important things for me without hesitation.	3.48	1.18	0.78
If I’m very depressed, I know who I can turn to.	3.44	1.19	0.72
*Loneliness* (“Thinking about the past year, how often have you felt…”)			
That you lack companionship?	1.86	0.72	0.73
Left out?	1.77	0.72	0.84
Isolated from others?	1.86	0.73	0.71
*Resilience* (“To what extent do the following statements apply to you personally?”)			
I can deal with whatever comes my way.	2.55	0.92	0.69
I believe I can achieve my goals, even if there are obstacles.	2.50	0.97	0.74
Under pressure, I stay focused and think clearly.	2.41	1.01	0.72
I think of myself as a strong person when dealing with life’s challenges and difficulties.	2.48	1.03	0.70
*COVID-19 worry* (“How worried are you about the impacts of coronavirus?”)			
On the health of your loved ones?	3.69	1.16	0.66
On your own mental well-being?	3.04	1.28	0.75
On the mental well-being of your loved ones?	3.31	1.21	0.92

Notes: *M* = mean; *SD* = standard deviation; FL = factor loading

The items were also found to converge on their assigned factors (Table 2), as the average variance extracted (AVE) values exceeded the cut-off value of 0.50 and the composite reliabilities for all factors ranged from 0.77 to 0.93, demonstrating good internal reliability (Bagozzi & Yi, 2012). The measurement model was also tested for discriminant validity using Fornell and Lacker’s (1981) AVE method and Bagozzi’s (1991) method. The correlations between the constructs were equal to or below 0.37; therefore, the square roots of the AVEs showed acceptable discriminant validity.

**Table 2:** Validity, Reliabilities, and Intercorrelations

	***M***	***SD***	**α**	**CR**	**AVE**	**1**	**2**	**3**	**4**	**5**
COVID-19 worry	3.35	1.04	0.82	0.83	0.62	0.79				
Social Support	3.45	0.97	0.77	0.77	0.53	0.07	0.73			
Problem gambling	0.61	0.80	0.93	0.93	0.72	0.16	-0.04	0.85		
Loneliness	1.83	0.61	0.80	0.80	0.58	0.30	-0.33	0.37	0.76	
Resilience	2.49	0.78	0.81	0.81	0.51	-0.05	0.51	-0.12	-0.36	0.72

Notes: *M* = Mean, *SD* = standard deviation, α = Cronbach’s alpha; CR = composite reliability, AVE = average variance extracted; construct correlations, square root of AVEs (on the diagonal)

## Results

The results of the hypotheses testing are shown in Table 3. The model fit was assessed through several indices, which indicated a good fit despite the high chi-square value (Schermelleh-Engel et al., 2003). The values of IFI, TLI, RFI, and CFI were clearly above 0.90 and ranged from 0.97 to 0.98; the value of RMSEA was 0.03, SRMR was 0.03, and the value of CMIN/DF was below the cut-off value of 3, which indicated good fit for the model (Hu & Bentler, 1999). The conceptual model accounted for 31% of the variance in *resilience*, 32% of the variance in *loneliness*, and 26% of the variance in *PGSI*. The results also supported most of the proposed hypotheses.

In line with H1, *social support* was negatively associated with *loneliness* (*β* = -0.23, SE = 0.02, t = -12.44, *p* < 0.001), meaning that individuals who reported higher social support felt less lonely. *COVID-19 worry* was positively associated with *loneliness* (*β* = 0.18, SE = 0.02, t = 10.22, *p* < 0.001), thus supporting H2. Both *social support* (*β* = 0.36, SE = 0.03, t = 13.53, *p* < 0.001) and *loneliness* (*β* = -0.23, SE = 0.04, t = -7.06, *p* < 0.001) were associated with *resilience*, supporting H3 and H4, respectively. Contrary to H5, *social support* had a positive yet small effect on *problem gambling* (*β* = 0.07, SE = 0.03, t = 2.56, *p* < 0.01), indicating that gamblers who had received social support were likelier to suffer from gambling problems. In line with H6, higher levels of *loneliness* predicted *problem gambling* (*β* = 0.37, SE = 0.04, t = 8.90, *p* < 0.001). *Resilience* or *COVID-19 worry*, however, had no statistically significant associations with *problem gambling*, leaving H7 and H8, respectively, unsupported.

Finally, we tested the control variables (not reported in the tables). Higher *education* was positively associated with *resilience* (*β* = 0.05, SE = 0.01, t = 4.16, *p* < 0.001). *Age* was negatively associated with *loneliness* (*β* = -0.01, SE = 0.01, t = -12.49, *p* < 0.001) and *problem gambling* (*β* = -0.02, SE = 0.01, t = -14.95, *p* < 0.001), indicating that younger adults have been more prone to experience loneliness and gambling problems during the pandemic. *Age* also had a positive association with *resilience* (*β* = 0.003, SE = 0.01, t = 3.24, *p* < 0.01), meaning that older adults were more likely to report higher psychological resilience compared to younger adults. *Female gender* was positively associated with *loneliness* (β = 0.09, SE = 0.02, t = 4.03, *p* < 0.001) and negatively associated with *problem gambling* (β = -0.20, SE = 0.03, t = 6.50, *p* < 0.001), indicating that women are more susceptible to loneliness, and men are more susceptible to gambling problems.

**Table 3:** Results of Hypotheses Testing

**DV**	**IV**	**Hypothesis**	**β**	**SE**	**t**	**R** ^2^
Loneliness	Social Support	H1: supported	-0.23***	0.02	-12.44	0.32
Loneliness	COVID-worry	H2: supported	0.18***	0.02	10.22	
Resilience	Social Support	H3: supported	0.36***	0.03	13.53	0.31
Resilience	Loneliness	H4: supported	-0.23***	0.04	-7.06	
PGSI	Social Support	H5: unsupported (reverse)	0.07**	0.03	2.56	0.26
PGSI	Loneliness	H6: supported	0.37***	0.04	8.90	
PGSI	Resilience	H7: unsupported	-0.03^ns^	0.03	0.08	
PGSI	COVID-worry	H8: unsupported	0.00^ns^	0.02	1.20	

Notes: ns = not significant, * = *p* < 0.05, ** = *p* < 0.01, *** = *p* < 0.001; DV = dependent variable, IV = independent variable, SE = standard error, t = t-value, R^2^ = R squared; Model fit: χ2 (165) = 460.61; CMIN/DF = 2.79; IFI = 0.98; CFI = 0.98; TLI = 0.98; RMSEA = 0.03; 90% CI [0.03, 0.04]; SRMR = 0.03; RFI = 0.97

## Discussion

The aim of this study was to examine the psychosocial factors associated with pandemic-time problem gambling. Drawing from our theoretical background, we approached loneliness and COVID-19 worry as potential risk factors and resilience and social support as potential protective factors. The findings mostly supported our hypotheses and previous studies, but contrary to expectations, we did not find evidence of the protective role of psychological resilience or social support in problem gambling.

As expected, loneliness was found to be associated with higher problem gambling severity, making it a potential risk factor for problem gambling. This aligns with previous studies (Khazaal et al., 2017; Sirola et al., 2019; Vuorinen et al., 2021), which emphasize that psychosocial problems often co-occur with addictive behaviors and that loneliness has adverse effects on well-being. As hypothesized, we also found that social support had a negative association with loneliness, indicating that individuals who have supportive social networks felt less lonely during the pandemic.

Contrary to expectations, there was a positive association between social support and problem gambling, indicating that social support might be a potential risk factor for problem gambling. This finding is in contrast to previous literature on the protective role of social support (Cohen & Wills, 1985; Mancini & Bonanno, 2009; Zhang et al., 2022), thus challenging the view of social support as a solely protective factor. Previous studies have yielded mixed findings regarding the role of social support in problem gambling (for a review, see Nordmyr & Forsman 2020). For example, studies on youth gambling have shown that support derived from online networks is particularly harmful compared to offline support (Savolainen et al., 2019) but also that social support from offline peers can increase problem gambling tendencies (Dowling et al., 2017; Räsänen et al., 2016; Yücel et al., 2015). Thus, some forms of social support might be riskier than others regarding problem gambling (Bush et al., 2021; Savolainen et al., 2019), but these risks might also work differently among different populations, such as adolescent gamblers. Indeed, gamblers and problem gamblers are not homogenous groups; different groups have varying vulnerabilities and risk factors (Blaszczynski & Nower, 2002; Sharman et al., 2019). It is also possible that some individuals with gambling problems report high levels of social support due their distress and elevated need for support. Therefore, it is important to study these effects in more detail.

COVID-19 worry was not directly associated with problem gambling. However, as hypothesized, COVID-19 worry was associated with loneliness, which in turn was associated with problem gambling. Thus, negative emotions, such as rumination, anxiety, and excessive worry regarding the pandemic’s impact, might be harmful and increase feelings of loneliness and isolation (Arslan et al., 2022; Hoffart et al., 2020), which might manifest in problematic behaviors such as problem gambling.

As hypothesized, social support and loneliness were associated with resilience, supporting the idea that those with meaningful social connections are more capable of coping during crisis situations (Cohen & Wills, 1985; Mancini & Bonanno, 2009; Zhang et al., 2022). While previous research has found that resilience is a protective factor, particularly in youth gambling problems (Lussier et al., 2007), we did not find evidence of this association for adult gamblers. A similar lack of protective factors among adult gamblers has also been noted in other studies (Mishra et al., 2019; Oei & Goh, 2015; Scholes-Balog et al., 2015). One reasonable explanation for this is that because many behavioral addictions start to develop during childhood or adolescence (Derevensky et al., 2019), protective factors might work differently among adults compared to younger individuals. As Scholes-Balog et al. (2015) argued, problem gambling tendencies often stem from adolescence; therefore, psychosocial factors, such as social support or psychological resilience, might not have relevant protective roles later in life. Thus, it is crucial to consider potential vulnerabilities and protective factors at a young age to prevent future problem behaviors and related negative consequences.

Regarding our control variables, it was found that male gender was associated with problem gambling, which aligns with previous studies (Dowling et al., 2017; Hing et al., 2016; Merkouris et al., 2016). Female participants reported more loneliness than males, which was recognized during the pandemic (Lee et al., 2020). Younger age was associated with both problem gambling and loneliness. Indeed, previous research has shown that problem gambling is most typical among young adults (Hing et al., 2016; Salonen et al., 2020), and loneliness and its negative effects are particularly prevalent among young people, such as emerging adults (Lee et al., 2020; Qualter et al., 2015).

Given our findings, particularly regarding the role of loneliness in problem gambling, it is important to consider strengthening meaningful social connections and providing beneficial support. However, social support is not always beneficial in decreasing loneliness. For example, in terms of addictive behaviors, such as problem gambling, a sense of loneliness can be derived from a perceived lack of understanding by loved ones or being alone with addiction or related problems while still receiving social support in other life areas. This kind of emotional loneliness can occur despite the quantity of social relationships (Weiss, 1973). Additionally, some problem gamblers might borrow money from loved ones to finance their gambling, thus gaining financial support, even if they conceal their gambling behavior from others and feel alone with their problems. Indeed, problem gamblers are often prone to concealing their problematic behaviors from their loved ones due to the stigma and shame associated with problem gambling (Hing et al., 2014), which might increase their sense of emotional loneliness.

Regarding problem gambling, it is also important to consider the risks of support derived from online communities, such as those dedicated to promoting and normalizing excessive gambling (Sirola et al., 2021). These kinds of communities have been acknowledged as risk factors for problem gambling, particularly among youth gamblers (Savolainen et al., 2022b; Sirola et al., 2021). During the COVID-19 pandemic, recognizing the role of online support is particularly important, given that digital technologies have been emphasized as a means to social interaction. Additionally, lonely individuals often use social media excessively to compensate for a lack of meaningful offline relationships and support (O’Day & Heimberg, 2021). Thus, particularly in terms of addictive behaviors, such as problem gambling, more focus should be placed on the quality of social support, such as whether it supports excessive behaviors or otherwise makes it possible to maintain harmful habits.

Finally, despite the cross-sectional nature of this study, our findings emphasize vulnerabilities regarding problem gambling. Given that individuals suffering from gambling problems have been recognized as a vulnerable group during the pandemic (Brodeur et al., 2021; Hodgins & Stevens, 2021), it is important to pay attention to these vulnerabilities in post-pandemic life and future crisis situations. Excessive gambling has the potential to burden financial well-being and lead to indebtedness and long-lasting problems (Oksanen et al., 2019). Moreover, problem gambling is consistently found to be associated with self-harm, such as suicidal ideation (Gray et al., 2021; Sundqvist & Wennberg, 2022), which underlines the importance of early intervention. While the pandemic has amplified psychosocial problems, such as loneliness, among already vulnerable individuals (Groarke et al., 2020; Lee et al., 2020; Luchetti et al., 2020), new problems and vulnerabilities might have occurred. For example, social distancing might have manifested in loneliness among those who had not felt lonely before, and consequently in maladaptive and excessive behaviors as a response to unideal situations. Despite the many benefits of digital technologies during social isolation, some individuals have also been more susceptible to developing harmful online habits (Király et al., 2020; Sirola et al., 2022). Developing problem behaviors during the pandemic might have long-lasting consequences in post-pandemic life, making it important to recognize potential risk and protective factors and to prevent future problems.

### Limitations

Some limitations must be acknowledged. First, cross-sectional data have limitations. Although our data were collected in April 2021 (i.e., approximately one year after the worldwide spread of the COVID-19 pandemic), and most of the survey questions were modified to concern pandemic-time experiences and behaviors, we were unable to assess how respondents’ gambling behaviors or other measured attributes might have changed compared to the time before the pandemic or over the course of the pandemic. Thus, our findings reflect only the first year of the pandemic, and the results should be interpreted accordingly. In addition, with cross-sectional data, all assumed causal directions between variables are purely theoretical (e.g., risk and protective factors), and studied associations between variables might also work vice versa. For example, addictions often strain social relationships (Griffiths, 2005) and may thus increase feelings of loneliness. Longitudinal studies are needed to explore causal directions and how these associations develop over time. Second, the survey comprised self-reported measures that might be prone to biases, particularly in terms of stigmatized behaviors such as problem gambling. However, the use of an anonymous online survey potentially lowered the threshold for reporting such activities. Third, regarding the social support measure, a source of support was not specified (e.g., offline vs. online, support coming from other gamblers, etc.). More detailed studies are needed to explore the quality and sources of social support and how these associations might work differently between different subpopulations of gamblers. Finally, our data were gathered from adult gamblers over 18 years of age, and the findings are not generalizable to the non-gambling population or adolescent gamblers. Given that adolescent problem gambling is a worldwide problem and that problem gambling patterns often initiate during adolescence (Calado et al., 2017; Derevensky et al., 2019), it would be highly important to focus on underaged gamblers in terms of risk and protective factors.

### Conclusion

The effects of the COVID-19 pandemic can be long lasting, making it crucial to study vulnerable groups, such as gamblers and problem gamblers, in the post-pandemic world. Problem gambling poses significant risks for strain on emotional, social, and financial well-being for a long period of time, making it important to recognize potential vulnerabilities and to create timely and effective interventions. The findings of this study emphasized psychosocial risks, namely, a lack of meaningful social connections in problem gambling during the pandemic. Thus, loneliness should be recognized as a major vulnerability in clinical settings, as it is destructive to well-being and might manifest in maladaptive behaviors such as problem gambling. However, given that our findings indicate that social support does not necessarily protect people from gambling problems and can even increase the risk, it would be crucial to examine in greater detail how different sources and quality of social support might work differently among different populations of gamblers.

## Data Availability

Data will be made available upon reasonable request.
